# A New Type of Endometrial Cancer Models in Mice Revealing the Functional Roles of Genetic Drivers and Exploring their Susceptibilities

**DOI:** 10.1002/advs.202300383

**Published:** 2023-06-20

**Authors:** Jingyao Chen, Siqi Dai, Lei Zhao, Yiman Peng, Chongen Sun, Hongling Peng, Qian Zhong, Yuan Quan, Yue Li, Xuelan Chen, Xiangyu Pan, Ailing Zhong, Manli Wang, Mengsha Zhang, Shengyong Yang, You Lu, Zhong Lian, Yu Liu, Shengtao Zhou, Zhengyu Li, Feifei Na, Chong Chen

**Affiliations:** ^1^ Precision Medicine Research Center State Key Laboratory of Biotherapy and Cancer Center West China Hospital Sichuan University Chengdu 610041 China; ^2^ State Key Laboratory of Biotherapy and Cancer Center West China Hospital Sichuan University Chengdu 610041 China; ^3^ West China Second Hospital Sichuan University Chengdu 610041 China; ^4^ Department of Dermatology State Key Laboratory of Biotherapy and Cancer Center National Clinical Research Center for Geriatrics West China Hospital Sichuan University Chengdu 610041 China; ^5^ Division of Thoracic Tumor Multimodality Treatment Cancer Center West China Hospital Sichuan University Chengdu 610041 China; ^6^ Laboratory of Clinical Cell Therapy, West China Hospital Sichuan University Chengdu 610041 China

**Keywords:** animal model, drug screening, endometrial cancer, organoid, organoid‐initiated precision cancer models

## Abstract

Endometrial cancer (EC) is the most common female reproductive tract cancer and its incidence has been continuously increasing in recent years. The underlying mechanisms of EC tumorigenesis remain unclear, and efficient target therapies are lacking, for both of which feasible endometrial cancer animal models are essential but currently limited. Here, an organoid and genome editing‐based strategy to generate primary, orthotopic, and driver‐defined ECs in mice is reported. These models faithfully recapitulate the molecular and pathohistological characteristics of human diseases. The authors names these models and similar models for other cancers as organoid‐initiated precision cancer models (OPCMs). Importantly, this approach can conveniently introduce any driver mutation or a combination of driver mutations. Using these models,it is shown that the mutations in *Pik3ca* and *Pik3r1* cooperate with *Pten* loss to promote endometrial adenocarcinoma in mice. In contrast, the *Kras* G12D mutati led to endometrial squamous cell carcinoma. Then, tumor organoids are derived from these mouse EC models and performed high‐throughput drug screening and validation. The results reveal distinct vulnerabilities of ECs with different mutations. Taken together, this study develops a multiplexing approach to model EC in mice and demonstrates its value for understanding the pathology of and exploring the potential treatments for this malignancy.

## Graphical abstract

This study reports an organoid and genome editing‐based strategy to generate primary, orthotopic, and driver‐defined EC models in mice. These models are named as organoid‐initiated precision cancer models (OPCMs). These EC models facilitate the functional studies of patients‐associated genetic alterations in tumorigenesis and provide precision models for preclinical drug testing.

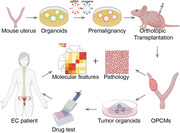

## 1 Introduction

Endometrial cancer (EC) is a malignancy of the inner epithelial lining of the uterus, with an increasing incidence and disease‐associated mortality, worldwide.^[^ ^1^ ^]^ It is the sixth most commonly diagnosed cancer in women, accounting for ≈30% of female reproductive tract cancers.^[^ ^2^ ^]^ Patients with stage III and above disease have high recurrence and metastasis rates after treatment, and the prognosis is extremely poor. For stage III and stage IV patients, the 5‐year overall survival rates are 57–66% and 20–26%, respectively.^[^ ^3^ ^]^ The current first‐line treatment for advanced or recurrent EC is systemic chemotherapy with carboplatin and paclitaxel, which were first introduced more than 20 years ago.^[^ ^4^ ^]^ Although some approved targeted drugs generally tend to have some activity in endometrial cancer, they have not been shown to have major benefits.^[^ ^4b^ ^]^ The utility of immune checkpoint inhibitors has been proven modest in advanced or recurrent ECs.^[^ ^5^ ^]^ Indeed, there are very few treatment options for patients with advanced and recurrent EC for whom first‐line therapies failed. Thus, there is an urgent need for new treatments that can precisely target tumor cells with fewer side effects for EC.

Recently, genomic analyses of large cohorts of EC patients have shown that deficiency of tumor suppressors such as *TP53*, *PTEN*, *BRAC2*, and *ARID1A*, and gain‐of‐function mutations or amplification of oncogenes like *ERBB2*, *KRAS, CCNE1*, and *MYC* are among the most frequent genetic abnormalities associated with EC.^[^ ^6^ ^]^ Based on the integrated genomic characterization of EC, Cyriac et al., creatively classified EC into four categories: POLE ultramutated, microsatellite instability hypermutated, copy number low, and copy number high.^[^ ^7^ ^]^ Interestingly, EC has more frequent mutations in the PI3K/AKT pathway than any other tumor type studied by TCGA. Several prior reports have revealed that *PTEN* mutations co‐exist frequently with other mutations in the PI3K/AKT pathway.^[^ ^8^ ^]^ Moreover, *KRAS* gain‐of‐function mutations are found in ≈20% of endometrioid endometrial carcinomas.^[^ ^9^ ^]^ And consistently, *KRAS* mutations frequently coexist with *PTEN* mutation, which activates independent events from PI3K pathway aberrations.^[^ ^8^, ^10^ ^]^ Nevertheless, due to the lack of available animal models of EC, our understanding of the potential functions and underlying mechanisms of these mutations in the initiation and progression of EC is still very limited, which hampers the development of genome‐guided treatments.

Although EC cell lines have significantly contributed to the basic and translational studies on EC, the lack of tumor heterogeneity and microenvironmental factors make them inadequate to represent this disease.^[^ ^11^ ^]^ Subsequently, patient‐derived xenografts (PDXs) and genetically engineered mouse models (GEMMs) have been generated.^[^ ^12^ ^]^ PDXs can maintain the genetic alterations and pathologic features of patient tissue, enabling them to be harnessed to assess the therapeutic potential of drugs.^[^ ^13^ ^]^ However, they cannot represent the whole transforming process of carcinogenesis in normal cells.^[^ ^14^ ^]^ GEMMs are also restricted for many reasons, including the lack of tissue‐specific Cre, high cost, and complex breeding processes.^[^ ^15^ ^]^ Recent progress in endometrial organoid culture has provided an advanced strategy for EC modeling.^[^ ^16^ ^]^ Organoids can recapitulate the cell heterogeneity, in vivo functions, and genetic characteristics of original tissues.^[^ ^17^ ^]^ Here, we report an organoid and genome editing‐based strategy to generate primary, orthotopic, and driver‐defined ECs in mice. These models faithfully recapitulate the molecular and pathohistological characteristics of human diseases. Importantly, this approach can conveniently introduce any driver mutation or combination of driver mutations to identify their biological functions in the initiation and progression of cancer.^[^ ^18^ ^]^ These organoid‐based models with defined driver mutations can precisely mimic the pathologies, molecular and cellular features, as well as treatment response, and thus, are named asorganoid‐initiated precision cancer models (OPCMs) to be distinct from GEMMs. And we also performed drug screening on these tumor organoids with different mutations and found that the organoids showed genomic mutation‐specific drug responses. This strategy opens the possibility to study the process of tumorigenesis and explore precise therapeutics for this disease.

## 2 Results

### 2.1 A Novel Primary Orthotopic Endometrial Cancer Generated with Genome‐Editing Endometrial Organoids in Mice

**Figure 1:**
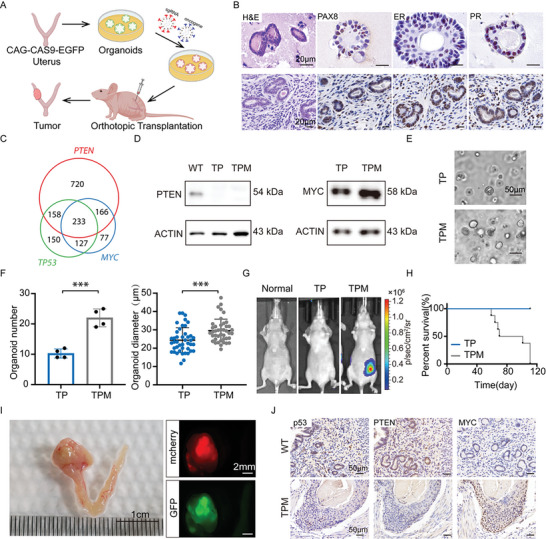
Primary and orthotopic endometrial cancers initiated with genome‐editing endometrial organoids in mice. A) The schematic of the organoid‐based strategy for generating primary and orthotopic EC in nude mice. B) H&E and IHC stainings of normal uterus organoid and tissue. Scale bar, 20 µm. C) Venn diagram showing 233 EC samples harbored all *PTEN*, *TP53*, and *MYC* alterations. EC patients’ data were analyzed from the cBioPortal dataset. D) Western blotting of PTEN and MYC of the TP and TPM samples. E) Representative bright field pictures of normal uterus organoids transduced by viral vectors, taken at 48 h after infection. Scale bar, 50 µm. F) Number of TP and TPM uterus organoids (left). Diameter of each organoid in TP and TPM groups, measured with Image J software (right). Data are shown as means ± SD. Significance was calculated by a two‐sided Student's *t*‐test. ***p <  0.001. G) Bioluminescent images of recipient mice one month post‐transplanted with TP and TPM uterus organoids. H) The Kaplan–Meier survival curve of recipient mice with TP and TPM uterus organoids. I) Representative bright‐field image of the uterus from moribund recipient mice (left). Showing the lesion on the left uterus. Representative fluorescent images of the lesion on the uterus (right). J) Representative IHC staining of p53, PTEN, and MYC in the TPM tumors and wild‐type tissues. Scale bar, 50 µm.

To mimic the pathology of human EC, we created a strategy to generate primary, orthotopic, and driver‐defined EC in mice with genome‐edited uterus organoids (**Figure** **1**A). Firstly, organoids were cultured from the normal uterus tissue of CAG‐Cas9‐EGFP mice,^[^ ^19^ ^]^ which were displayed as hollow spheres with smooth surfaces and grew rapidly. Histological analyses revealed that the organoids faithfully recapitulated the histological characteristics of the original tissues (Figure 1B and Figure S1A, Supporting Information). Markers of the glandular epithelium (CK7 and PAX8) were strongly expressed in organoids (Figure 1B and Figure S1A, Supporting Information). Estrogen receptor (ER) and progesterone receptor (PR) are present in the endometrial glandular epithelium of tissue and play a crucial role in hormone response.^[^ ^20^ ^]^ Histologically, the organoids exhibited a mosaic pattern of ER and PR staining (Figure 1B). Furthermore, we tested whether mouse uterus organoids could represent the molecular features of the endometrial epithelium by transcriptomics analyses. Gene signature enrichment analyses (GSEA) showed that the upregulated and downregulated genes in the human endometrial epithelium were significantly positively and negatively, respectively, enriched in mouse uterus organoids, compared to other organ organoids (Figure S1B, Supporting Information). Thus, mouse uterus organoids could mimic both cellular composition and molecular features of the endometrial epithelium.

Then, we analyzed the genetic landscape of 1954 EC samples from the cBioPortal dataset (https://www.cbioportal.org/). *PTEN* was one of the most frequently altered genes, which was disrupted in up to 66% of EC samples. *TP53* was also among the most frequently altered gene in human EC, with a mutation ratio of 34% (Figure S1C, Supporting Information). About 24% of all EC samples harbored *TP53* and *PTEN* variations together (Figure 1C and Figure S1C, Supporting Information). *MYC* was amplified in 31% of samples (Figure S1C, Supporting Information). The high expression level of *MYC* was also associated with poor prognosis of EC patients (Figure S1D, Supporting Information). Remarkably, *MYC* amplification tended to co‐occur with *PTEN* and *TP53* mutations (Figure 1C and Figure S1C, Supporting Information). Therefore, to test the roles of these genes in endometrial tumorigenesis, we introduced sgRNAs targeting *Trp53* and *Pten* into uterus organoids derived from CAG‐Cas9‐EGFP mice with a lentivirus vector (V2TC) carrying mCherry as a tracking marker (Figure S1E,F, Supporting Information). Meanwhile, *Myc* was overexpressed together with luciferase to facilitate in vivo monitoring of these organoids after transplantation (TPM) (Figure S1E, Supporting Information). The T7E1 assay demonstrated the successful mutation of *Trp53* and *Pten* and the luciferin assay showed the overexpression of *Myc* in organoids (Figure S1G,H, Supporting Information). We introduced empty vector‐linked luciferase, together with *Trp53* and *Pten* sgRNAs in uterus organoids, as control (TP). The decreased level of PTEN in TP and TPM organoids and the increased level of MYC in TPM organoids were confirmed by Western blotting (Figure 1D). TPM organoids displayed significantly increased size and number compared to TP organoids (Figure 1E,F). To test the in vivo tumorigenic capacity of TP and TPM organoids, we orthotopically transplanted them into nude mice (Figure S1I, Supporting Information). TPM organoids survived and grew over time, as indicated by the *Myc*‐linked luciferase bioluminescent image (Figure 1G). The recipient mice transplanted with TPM organoids developed tumors with an average latency of 85 days, while none of the recipient mice with TP organoids did in the observation period (Figure 1H). All TPM mice died with lesions at the organoid‐transplant sites on their left uterus. The resulting tumors were both mCherry and EGFP positive, indicating that they were derived from the transplanted TPM organoids (Figure 1I). Consistently, immunohistochemistry (IHC) staining confirmed the downregulation of p53 and PTEN expression and the upregulation of MYC in TPM tumors compared to that in wild‐type uterus tissue (Figure 1J). Thus, we demonstrated that uterus organoids with EC‐associated mutations and amplification were able to initiate malignancies in mice.

### 2.2 Disruption of *Trp53* and *Pten*, together with *Myc* Overexpression Generated Endometrial Adenocarcinoma with Squamous Differentiation

**Figure 2:**
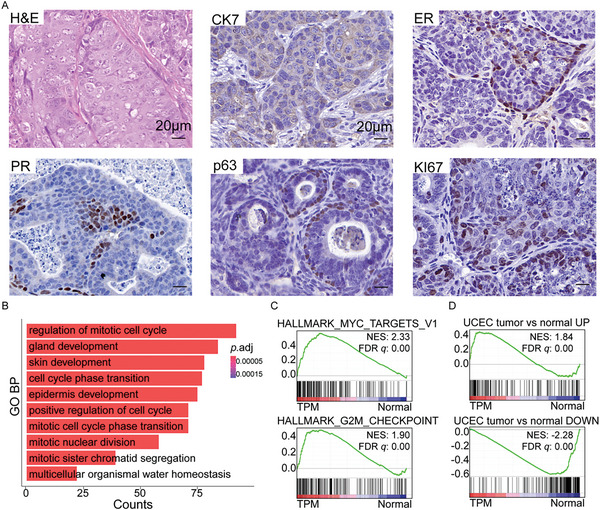
Disruption of *Trp53* and *Pten*, together with *Myc* overexpression generated endometrial adenocarcinoma with squamous differentiation. A) H&E and IHC staining of the uterus section of mice transplanted with the TPM uterus organoids. Scale bar, 20 µm. B) GO enrichment plot of the upregulated genes in the TPM tumors compared to normal endometrium. C) GSEA showing positive enrichments of the HALLMARK_MYC_TARGETS_V1 (NES = 2.33, FDR *q* = 0.00), and the HALLMARK_G2M_CHECKPOINT (NES = 1.90, FDR *q* = 0.00) signatures in the TPM tumors, compared to normal tissues. D. GSEA showing the enrichments of gene signatures (top 200 DEGs of upregulated or downregulated, *p*‐value < 0.05) of endometrial carcinoma patients (from TCGA‐UCEC) in the TPM murine EC tissue, compared to normal (UP: NES = 1.84, FDR *q* = 0.00; DOWN: NES = −2.28, FDR *q* = 0.00).

Histological analyses showed that the lesions contained glandular structures and were mostly composed of tumor cells with scant cytoplasm (**Figure** **2**A). And IHC staining showed that the TPM tumors were CK7 positive, indicating their epithelial origin (Figure 2A). Some of the tumor cells were hormone receptor‐positive (ER+, PR+), indicating their ability to respond hormonally (Figure 2A). The tumors also expressed the squamous marker p63 and had keratin pearls and concentric layers of keratin deposition which were pink with hematoxylin and eosin (H&E) staining, displaying features of squamous differentiation (Figure 2A). KI67, a proliferation marker, was highly expressed, confirming that it is an aggressive EC (Figure 2A).

To understand the molecular characteristics of the TPM murine EC, the transcriptome of EC tissues was compared to that of normal endometrium tissues by RNA‐seq. The heatmap of differentially expressed genes (DEGs) suggested that TPM tumors were distinct from normal endometrium tissues. Among the DEGs, multiple squamous differentiation‐related genes, including *Krt17*, *Krt8*, and *Trp63*, were upregulated in the TPM tumors (Figure S2A, Supporting Information). Consistently, both Gene Ontology (GO) analysis and GSEA showed that multiple gene sets related to keratinization and squamatization were significantly positively enriched in the TPM tumors compared to normal tissues (Figure 2B and Figure S2B, Supporting Information). In the majority of human endometrial tumors, estrogen signaling acts as an oncogenic signal.^[^ ^21^ ^]^ In the TPM murine EC, the HALLMARK_ESTROGEN_RESPONSE_EARLY and the HALLMARK_ESTROGEN_RESPONSE_LATE were significantly positively enriched, suggesting that the TPM tumors were the estrogen‐dependent EC (Figure S2B, Supporting Information). Further, GO analysis showed that multiple pathways related to the cell cycle were upregulated in the TPM tumors compared to normal tissue (Figure 2B). And also, GSEA revealed that multiple signatures related to cell cycle and malignant proliferation, including the HALLMARK_MYC_TARGETS_V1, the HALLMARK_G2M_CHECKPOINT, the HALLMARK_P53_PATHWAY, and the HALLMARK_E2F_TARGETS, were significantly positively enriched in the TPM tumors compared to normal tissue (Figure 2C and Figure S2B, Supporting Information). Hence, these results strongly suggested that the TPM tumors were endometrial adenocarcinoma with squamous differentiation.

Furthermore, GSEA showed that mouse EC derived from organoids could represent the molecular features of EC patients at the transcriptome level. The upregulated and downregulated genes in the EC patients were significantly positively and negatively, respectively, enriched in mouse EC, compared to mouse normal tissue (Figure 2D). These results strongly suggested that the TPM tumors recapitulated the features of their human counterpart.

### 2.3 *Pik3ca* and *Pik3r1* Mutations Accelerated the Progression of TPM Tumors

**Figure 3:**
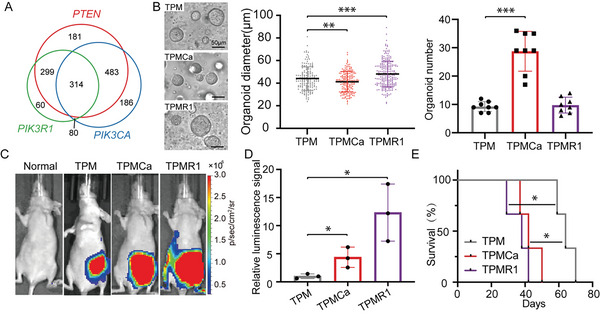
*Pik3ca* and *Pik3r1* mutations accelerated the progression of TPM tumors. A) Venn diagram showing the intersection of the human EC with PTEN, *PIK3R1*, and *PIK3CA* mutations. *p*‐value (*PTEN* and *PIK3R1*) = 8.3e‐34, *p*‐value (*PTEN* and *PIK3CA*) = 1.1e‐22, and *p*‐value (*PIK3CA* and *PIK3R1*) = 0.9. EC patient data were analyzed from the cBioPortal dataset. Statistic values were determined by a hypergeometric test. B) Representative bright field pictures of normal uterus organoids transduced by viral vectors. Scale bar, 50 µm (left). Diameters of the organoids in the premalignant stage. (TPM: *n* = 157; TPMCa: *n* = 176; TPMR1: *n* = 229) (middle). Number of TPM, TPMCa, and TPMR1 uterus organoids (right). Data shows the means ± SD. **, *p* < 0.01, ***, *p* < 0.001, calculated by two‐sided Student's *t*‐test. C) Bioluminescent images of recipient mice one month post‐transplanted with uterus organoids. D) The luminescence signal intensity of the TPM, TPMCa, and TPMR1 mice. Data shows the means ± SD. *, *p* < 0.05, calculated by two‐sided Student's *t*‐test. E) Kaplan–Meier survival curves of mice transplanted with the TPM, TPMCa, and TPMR1 organoids. All curves were analyzed by log‐rank test.

Previous meta‐analyses have shown that phosphatidylinositol 3‐kinase (PI3K) pathway abnormalities occur at a high frequency in EC, which appears to be caused by PTEN protein loss.^[^ ^8b^ ^]^ *PIK3CA* and *PIK3R1*, as key regulators of the PI3K pathway, are frequently mutated in EC (Figure S3A, Supporting Information). To investigate the relationship between these factors, we also analyzed the cBioPortal dataset with a total of 1954 samples mentioned above. The results showed that 797 out of the total 1277 *PTEN* mutation samples had *PIK3CA* mutation, and 613 out of the total 1277 *PTEN* mutation samples had *PIK3R1* mutation (**Figure** **3**A and Figure S3A, Supporting Information), suggesting that *PTEN* mutation was associated with *PIK3CA* mutation and *PIK3R1* mutation in human EC. However, the mutation events statistics for *PIK3CA* and *PIK3R1* per sample showed that they were exclusive (Figure 3A and Figure S3A, Supporting Information). The main genetic alterations of the *PIK3CA* gene were copy number variations (amplification and gain) and missense mutations (Figure S3A, Supporting Information). And, high *PIK3CA* expression was associated with poor prognosis in patients with EC (Figure S3B, Supporting Information). For the *PIK3R1* gene, shallow deletion accounted for the majority of its alterations (Figure S3A, Supporting Information). Further, the low expression level of the *PIK3R1* gene was associated with poor prognosis of EC patients (Figure S3C, Supporting Information). To better understand the biological functions and molecular mechanisms of these co‐mutations in this disease, we established and assayed EC models with these genetic alterations. The *PIK3CA* hotspots p.E545K and p.H1047R are the two most commonly reported mutated *PIK3CA* sites in endometrial cancer.^[^ ^10^ ^]^ Thus, *Pik3ca* E545K cDNA was overexpressed by a retrovirus, together with *Trp53* and *Pten* sgRNAs and *Myc* overexpression (TPMCa). Also, we transduced sgRNA targeting *Pik3r1*, together with *Trp53*, *Pten* sgRNAs, and *Myc* overexpression, into the primary uterus organoids (TPMR1). The Integrative Genomics Viewer (IGV) showed c.1633G > A mutation in the *Pik3ca* gene and the T7E1 assay demonstrated that *Pik3r1* has been disrupted (Figure S3D,E, Supporting Information). 48 h after infection, we took bright‐field images for each well of TPM, TPMCa, and TPMR1 groups (Figure 3B). In vitro, the number of TPMCa organoids was significantly more than that of TPM organoids, and the diameter of TPMCa was smaller than that of TPM organoids (Figure 3B). For the TPMR1 group, there was not a significant increase in organoid number compared with the TPM group and the diameter of TPMR1 organoids was significantly larger than that of TPM organoids (Figure 3B). To compare the in vivo tumorigenic capacity of TPM, TPMCa, and TPMR1 premalignant organoids, we orthotopically transplanted them into nude mice. Bioluminescent images of recipient mice with TPM, TPMCa, and TPMR1 organoids were performed at the same time (one month after transplantation). The results revealed that there was a significantly stronger luminescence signal on the left abdomen of both the recipient mice with TPMCa and TPMR1 organoids than that in the control TPM mice (Figure 3C,D). All of these recipient mice died with tumors at the organoid‐transplant sites on their left uterus. The TPMCa organoid recipient mice died with a significantly shorter latency (43 days) than the control TPM recipients. Similarly, all recipients of TPMR1 organoids had significantly shorter latency (36 days) (Figure 3E). Taken together, these data demonstrated that both *Pik3ca* E545K amplification and *Pik3r1* deficiency promoted EC tumorigenesis.

### 2.4 *Pik3ca* and *Pik3r1* Mutations were Dominant in the Endometrial Adenocarcinoma

**Figure 4:**
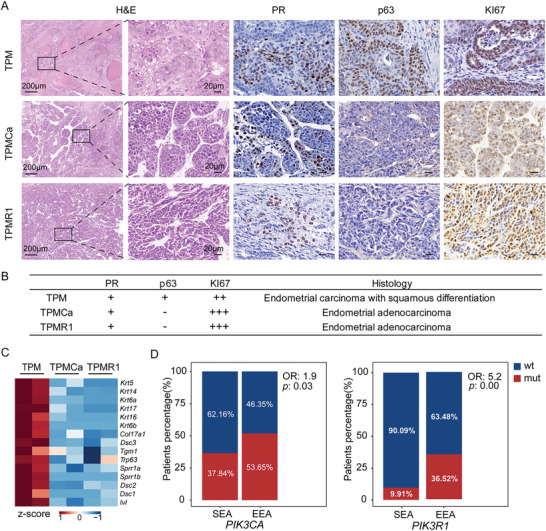
*Pik3ca* and *Pik3r1* mutations were dominant in the endometrial adenocarcinoma. A) Representative pictures showing H&E and IHC stainings of PR, p63, and KI67 in tumor sections of the TPM, TPMCa, and TPMR1 mice. Scale bar, 200 and 20 µm. B) IHC score of PR, p63, and KI67 of the TPM, TPMCa, and TPMR1 mice. C) Heatmap showing the expression levels of squamous marker genes between TPM, TPMCa, and TPMR1 mice tumor tissues (TPM: *n* = 2 mice; TPMCa: *n* = 2 mice; TPMR1: *n* = 2 mice). D) The percentage of SEA and EEA patients with *PIK3CA* or *PIK3R1* mutations. (*PIK3CA*: OR = 1.9, *p* = 0.03; *PIK3R1*: OR = 5.2, *p* = 0.00. Calculated by Fisher's test).

Firstly, IHC staining revealed that PIK3CA was strongly positive in TPMCa tumors, while PIK3R1 is negative in TPMR1 tumors, indicating that the tumor cells originate from a specific genotype of organoids (Figure S4A,B, Supporting Information). Then we performed histological analyses of the resulting tumors. H&E staining revealed that TPMCa and TPMR1 tumors contained well‐differentiated glandular structures without keratin pearls, in contrast to the adenocarcinoma with the squamous feature of the TPM tumors (**Figure** **4**A,B). Consistently, p63 was negative in the TPMCa and TPMR1 tumors (Figure 4A,B). IHC staining revealed the increased expression level of KI67 in the TPMCa and TPMR1 tumors compared to the TPM tumor. Similar to the TPM tumors, PR was only slightly expressed in the TPMCa and TPMR1 tumors (Figure 4A,B). To characterize the molecular features of these tumors, we performed transcriptome analyses of the TPM, TPMCa, and TPMR1 tumor tissues. RNA‐seq data revealed that the TPMCa and TPMR1 tumors were clearly separated from the TPM tumor by PCA (Figure S4C, Supporting Information). And the heatmap suggested that the expressions of multiple squamous differentiation‐related genes decreased in the TPMCa and TPMR1 tumors (Figure 4C). GO pathways significantly negatively enriched in the TPMCa and TPMR1 groups were related to skin development, epidermis development, and keratinocyte differentiation (Figure S4D, Supporting Information). Consistent with p63 IHC staining, the expression of *Trp63* was significantly downregulated in the TPMCa and TPMR1 tumors compared to that in the TPM tumors (Figure S4E, Supporting Information). And further, GSEA revealed that keratinization‐related signaling pathways were significantly downregulated in the TPMCa and TPMR1 tumors compared to the TPM tumor (Figure S4F, Supporting Information). The molecular events downstream of PI3K lead to the activation of mTORC1.^[^ ^22^ ^]^ GSEA showed that mTORC1 signaling was significantly positively enriched in the TPMCa and TPMR1 tumors compared to the TPM (Figure S4F, Supporting Information). And also, consistent with KI67 IHC staining, GSEA revealed that the HALLMARK_E2F_TARGETS and the HALLMARK_G2M_CHECKPOINT were significantly positively enriched in the TPMCa and TPMR1 tumors, suggesting their increased cell proliferation (Figure S4F, Supporting Information). In addition, we performed the fisher‐test of *PIK3CA* or *PIK3R1* mutation events and histological subtypes in the TCGA‐UCEC cohort to confirm the dominance of *PIK3CA* and *PIK3R1* mutations in different EC subtypes. The results showed that both *PIK3CA* mutations and *PIK3R1* mutations were associated with endometrial endometrioid adenocarcinoma (EEA), compared with serous endometrioid adenocarcinoma (SEA) (OR = 1.9, 5.2 and *p* = 0.03, 0.00) (Figure 4D). These data suggested that the TPMCa and TPMR1 tumors exhibited classic pathological features of endometroid adenocarcinoma, in contrast to the TPM tumors, suggesting that *Pik3ca* and *Pik3r1* mutations switched the tumor types.

### 2.5 *Kras* G12D Mutation led to Endometrial Squamous Cell Carcinoma in Mice

**Figure 5:**
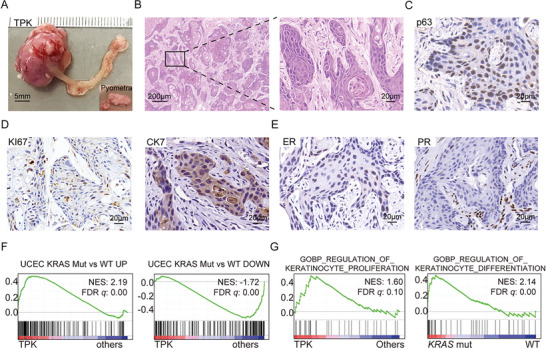
*Kras* G12D mutation led to endometrial squamous cell carcinoma in mice. A) Bright‐field (BF) of the uterus from moribund recipient TPK mice. Scale bar, 5 mm. Box areas showing pus in the uterus cavity. B) H&E stainings in the tumor section of the TPK mice. Scale bar, 200 µm (left) and 20 µm (right). C) Representative pictures showing staining of p63 in tumor sections of the TPK mice. Scale bar, 20 µm. D) Representative pictures showing staining of KI67 and CK7 in tumor sections of the TPK mice. Scale bar, 20 µm. E) Representative pictures showing staining of ER and PR in tumor sections of the TPK mice. Scale bar, 20 µm. F) GSEA showing the enrichments of gene signatures (top 200 DEGs of upregulated or downregulated, *p*‐value < 0.05) of human EC with *KRAS* mutation in TPK mice, compared to other models (TPM, TPMCa, and TPMR1) (UP: NES = 2.19, FDR *q* = 0.00; DOWN: NES = −1.72, FDR *q* = 0.00). G) GSEA showing the enrichments of the keratinocyte‐related pathway both in TPK mice (the GOBP_REGULATION_OF_KERATINOCYTE_PROLIFERATION, NES = 1.60, FDR *q* = 0.10) and human EC with *KRAS* mutation (the GOBP_REGULATION_OF_KERATI‐NOCYTE_DIFFERENTIATION, NES = 2.14, FDR *q* = 0.00), compared with other models or human EC with *KRAS* WT.

There is accumulating evidence suggesting that *KRAS* G12D is frequently activated in EC.^[^ ^10^ ^]^ Further, large‐scale genome sequencing studies indicated that *KRAS* G12D mutation and *MYC* amplification often were mutually exclusive (Figure S5A, Supporting Information). *KRAS* mutations frequently coexist with *PTEN* and *TP53* mutations (Figure S5B, Supporting Information). In patients with *TP53* and *PTEN* variation, a high expression level of *KRAS* was associated with poor prognosis (Figure S5C, Supporting Information). Recent studies suggested that *Kras* G12D played an oncogenic role in EC.^[^ ^23^ ^]^ It has been reported that *Kras* G12D organoids with *Trp53* deficiency developed subcutaneous carcinosarcoma. To establish orthotopically, *Kras* G12D‐driven EC and study the potential functions of this mutation in EC initiation and progression, we amplified *Kras* G12D cDNA, together with *Trp53* and *Pten* loss, into mouse uterus organoids (TPK). About 3 months after orthotopic transplantation, all recipients died and had tumor lesions on their left uterus. Unlike solid tumors as described above, TPK tumors displayed a sac‐like structure filled with pus, a condition known as pyometra (**Figure** **5**A). The IGV showed c.35C > T mutation in the *Kras* gene (Figure S5D, Supporting Information). IHC stainings of TPK tumor revealed the downregulation of p53 and PTEN expression and the upregulation of KRAS expression at protein levels (Figure S5E, Supporting Information). H&E staining revealed that TPK tumors displayed distinct histology, characterized by large areas of keratinization (Figure 5B). None of the sections showed any evidence of adenocarcinoma. IHC staining of the squamous marker p63 was significantly positive in TPK tumor cells, confirming the squamous differentiation of the tumors (Figure 5C). They were also positive for KI67 staining, suggesting that they were aggressive cancers (Figure 5D). IHC staining showed that the tumor cells were CK7+, indicating their glandular epithelial origin (Figure 5D). The ER and PR were weakly positive in TPK tumors (Figure 5E). Taken together, histological and IHC analyses suggested that these murine EC were initiated with TPK organoids as endometrial squamous cell carcinoma (ESCC). A key clinical feature of ESCC patients is pyometra, which was observed in the TPK mouse EC model (Figure 5A). To characterize the molecular features of these TPK models, we performed RNA‐seq analyses. Consistent with pathological features of TPK tumors, the heatmap and GO analysis showed that multiple squamous differentiation‐related genes and pathways were significantly positively enriched in TPK tumors compared to adenocarcinoma TPMCa and TPMR1 tumors (Figure S5F,G, Supporting Information). GSEA showed that the upregulated and downregulated gene sets in human EC with *KRAS* mutation were significantly positively and negatively, respectively enriched in the TPK model, compared with other models (Figure 5F). EC patients with *KRAS* mutation also shared common keratinocyte‐related pathways with TPK mice (Figure 5G). Hence, the TPK tumor mimicked human ESCC at both the pathological and molecular levels. In patients, ESCC is rare and its pathogenesis remains obscure.^[^ ^24^ ^]^ According to previous reports, ESCC has a poorer prognosis than endometrioid carcinomas. To date, no final treatment recommendations have been provided. In the present study, we generated a primary endometrial squamous cell carcinoma in mice with *Kras* G12D uterus organoids.

### 2.6 EC Models for Drug Screening

**Figure 6:**
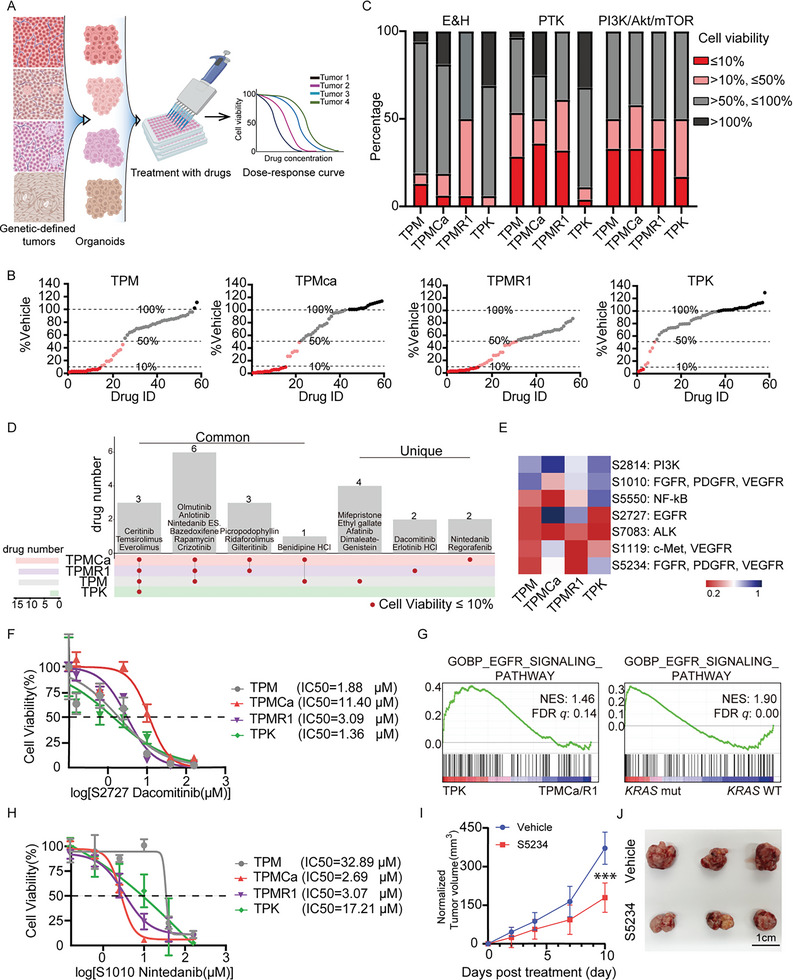
Precision EC models for drug screening. A) Schematic of the high‐throughput drug screening. Endometrial cancer cells derived from mouse models were cultured with Matrigel in 96‐well plates. After 24 h of culture, organoids were treated with compounds from a library. After 72 h of treatment, cell viability was analyzed by CCK8 assay. B) Summary of the drug library screening showing the relative survival ratios of the TPM, TPMCa, TPMR1, and TPK tumor organoids treated with individual drugs. The red dots indicated drugs‐inducing cell viability at 0–10%. The pink dots indicated drugs inducing cell viability at 10–50%. The gray dots indicated drugs inducing cell viability at 50–100%. The black dots indicated drugs inducing cell viability at more than 100%. C) Incidence of cell viability at 0–10%, 10–50%, 50–100%, and more than 100% after treatment in each kind of target. D) Upset plot showing the common and unique drug inducing cell viability at 0–10% in the TPM, TPMCa, TPMR1, and TPK EC organoids. Red dot, drugs inducing cell viability at 0–10%. E) Heatmap showing the relative survival of EC organoids treated with seven drugs in secondary drug screening at the concentrations of 10 µM. Three independent repeats were performed. F) Drug dose‐response curve showing cell viability of the TPM, TPMCa, TPMR1, and TPK EC organoids in response to the treatment of dacomitinib (S2727). IC50 was shown. *n* = 3. G) GSEA showing the enrichments of the GOBP_EGFR_SIGNALING_PATHWAY both in TPK mice (NES = 1.46, FDR *q* = 0.14) and human EC with *KRAS* mutation (NES = 1.90, FDR *q* = 0.00), compared with TPMCa/TPMR1 mice or human EC with *KRAS* WT. H) Drug dose‐response curve showing cell viability of the TPM, TPMCa, TPMR1, and TPK EC organoids in response to the treatment of nintedanib (S1010). IC50 was shown. *n* = 3. I) Normalized tumor volume of the TPMR1 subcutaneous tumor with vehicle or S5234. Normalized tumor volume indicates the increased volume when the tumor volume at day 0 (at the start of treatment) is set to 0. *n* = 4. Data shows the means ± SEM. ***, *p* < 0.001, calculated by two‐sided Student's *t*‐test. J. Images of the subcutaneous tumors treated with vehicle or S5234 (representative of *n* = 3 mice). Scale bars, 1 cm.

Endometrial cancer organoids (ECOs) could be cultured from these tumors to facilitate in vitro drug treatment studies (Figure S6A, Supporting Information). To assess whether our ECOs were useful for in vitro drug sensitivity testing, we first generated dose‐response curves for carboplatin, the first‐line chemotherapy drug for EC, and calculated the half‐maximal inhibitory concentration (IC50). Carboplatin induces cell death in various ECOs with different sensitivities. Notably, TPK (IC50 = 46.19 µM) had a higher IC50 than TPM (IC50 = 22.09 µM), TPMCa (IC50 = 19.97 µM), and TPMR1 (IC50 = 10.37 µM) tumors (Figure S6B, Supporting Information). Multiple previous studies have demonstrated that cancer organoids respond to drugs based on their genomic alterations.^[^ ^25^ ^]^ High‐throughput screening has been extensively used in precision medicine for cancer treatment. Next, we used the previously reported screening platform^[^ ^26^ ^]^ to identify potential drugs based on these mutations‐defined EC organoids (**Figure** **6**A). A total of 56 compounds, targeting endocrinology & hormones (E&H), protein tyrosine kinase (PTK), and PI3K/AKT/mTOR, were screened on our organoid‐based screening platform (Figure S6C, Supporting Information). The TPM, TPMCa, TPMR1, and TPK organoids were respectively cultured in 96‐well plates. Each inhibitor was added to the wells at a concentration of 10 µM. Three days later, the survival of the organoids was measured by Cell Counting Kit‐8 (CCK8) and cell viability scores were calculated according to the percentages of CCK8 reads normalized to those treated with vehicle for each molecule. Among the libraries, only three molecules inhibited the growth of the TPK ECOs by more than 90% (cell viability score ≤ 10%), whereas the number of molecules in the TPM and TPMR1 was 14, and that in the TPMCa was 15 (Figure 6B). 21 out of 56 drugs had no inhibitory effect on TPK tumor organoids (cell viability score > 100%), indicating that TPK ECOs displayed high resistance to these drugs (Figure 6B). To determine the effect of genetic alterations on drug response, we compared the responses of the four ECOs to drugs targeting different pathways (Figure 6C and Figure S6D, Supporting Information). We found that half of the 16 drugs targeting E&H induced less than 50% cell viability in TPMR1 ECOs compared with TPM (3/16), TPMCa (3/16), and TPK (1/16), indicating that TPMR1 ECOs were much more sensitive to the drugs targeting E&H than the other three organoids (Figure 6C). Previous studies have suggested that alterations in the PI3K/AKT/mTOR pathway are strongly implicated in endometrial cancer pathogenesis, and targeting the effectors of this pathway is a rational therapeutic approach.^[^ ^27^ ^]^ We transduced genetic alterations of the key genes in the PI3K/AKT/mTOR pathway in these four EC tumors, including *Pten* deficiency, *Pik3r1* deficiency, and *Pik3ca* E545K amplification. As expected, there were no significant differences in the sensitivity of the four ECOs to the drugs targeting the PI3K/AKT/mTOR pathway (Figure 6C). Here, it was intriguing that TPK ECOs displayed lower sensitivity to drugs targeting the PTK pathway, compared to the other three ECOs (Figure 6C), consistent with the resistance of *Kras* mutant cancers to targeted therapies as previously reported.^[^ ^28^ ^]^ The mechanism of *Kras* mutation‐induced drug resistance in EC requires further study. The drugs that induced less than 10% cell viability in the four ECOs were listed and classified according to the commonness and specificity of each group, further suggesting the similarities and differences in drug responses induced by genetic alterations (Figure 6D). To further evaluate the translational potential of our mouse organoid platform, we established organoid lines from two patients with EC. Genetic sequence and pathological analysis revealed that these two tumor samples lacked mutations in the *KRAS* gene and exhibited pathological features of adenocarcinoma, similar to the TPMCa and TPMR1 mouse tumor models (Figure S6E–G, Supporting Information). As expected, treatment experiments revealed that the patient‐derived organoids (PDOs) were resistant to drugs such as S1120 and S2221(Figure S6F,G, Supporting Information). In contrast, drugs such as S5234, S7083, and S2128 significantly inhibited the growth of the PDOs, consistent with the results observed in mouse organoids(Figure S6F,G, Supporting Information). Hence, EC PDOs showed a pattern of drug sensitivities similar to the mouse model.

Further, we performed the second screening and validation experiments to confirm the inhibitory effect of some drugs on the four ECOs (Figure 6E). S2727, also named dacomitinib, is an approved first‐line therapy for metastatic, EGFR‐mutant non–small cell lung cancer (NSCLC). In phase 1 clinical study, it has been reported that the combination of dacomitinib and PD‐0325901 showed antitumor activity in *KRAS*‐mutation‐positive colorectal, pancreatic cancer, and NSCLC.^[^ ^29^ ^]^ Similarly, the second screening demonstrated that dacomitinib inhibited the in vitro growth of *Kras*‐mutated EC organoids which displayed extensive resistance to these screened drugs. To further test the in vitro inhibitory effects of dacomitinib on the four ECOs, we performed a validation experiment and calculated their IC50 respectively. The results showed that the TPK ECOs were more sensitive (IC50 = 1.36 µM) to dacomitinib than the TPMCa (IC50 = 11.40 µM) and TPMR1 (IC50 = 3.09 µM) ECOs (Figure 6F). GSEA revealed that EGFR target pathways such as the GOBP_EGFR_SIGNALING_PATHWAY were significantly enriched in the TPK tumors and the *KRAS* mutated human EC than in the TPMCa and TPMR1 that were resistant to dacomitinib, and *KRAS* WT human EC (Figure 6G).

Nintedanib, an intracellular inhibitor of tyrosine kinases, has been tested in phase II evaluations for the treatment of recurrent or persistent endometrial cancer.^[^ ^30^ ^]^ The validation experiment showed that nintedanib inhibited the growth of ECOs in a dosage‐dependent manner. Similar to the results of screening, we found that the TPMCa and TPMR1 ECOs were more sensitive (IC50 = 2.69 µM and IC50 = 3.07 µM, respectively) to nintedanib than the TPM (IC50 = 32.89 µM) and TPK (IC50 = 17.21 µM) organoids (Figure 6H). To explore the molecular mechanisms underlying the distinct suppression of the four ECOs, GSEA was performed to compare the gene signaling associated with the drug target gene *VEGFR*. EC patients with *PIK3CA* or *PIK3R1* mutation shared common VEGF pathways associated with the S1010 target, such as the BIOCARTA_VEGF_PATHWAY (Figure S6H, Supporting Information). Interestingly, the up‐regulated genes in TPMCa and TPMR1 compared with TPM were better overlapped with BIOCARTA_VEGF_PATHWAY than down‐regulated genes, indicating that TPMCA and TPMR1 tumor also had a better BIOCARTA_VEGF_PATHWAY enrichment than TPM (Figure S6I, Supporting Information). To test the potential effect of Nintedanib in vivo, we generated a subcutaneous endometrial cancer mouse model by injecting TPMR1 tumor cells into the 8‐week‐old nude mice. Given the solubility of the drug, we opted to conduct in vivo treatment using nintedanib ethanesulfonate salt (S5234). Mice were treated three times per week by oral gavage with H_2_O or 50 mg kg^−1^ nintedanib ethanesulfonate salt for ten days. Consistent with the in vitro inhibitory effect, the tumor growth of the treatment group was significantly repressed, compared to the H_2_O‐treated control group (Figure 6I,J). Taken together, these results supported organoids as preclinical models for the drug testing of tumors with different genetic alterations.

## 3 Discussion

Although EC is one of the most common gynecological malignancies, our understanding of the mechanisms underlying its tumorigenesis and translational studies remains very limited. One of the major obstacles might be the lack of proper animal models that precisely recapitulate the molecular and clinical features of human EC. Currently, chemically induced EC animal models and GEMMs of EC are mostly used in vivo, and few of them can fully recapitulate the entire process of EC in patients.^[^ ^12^, ^31^ ^]^ Based on GEMMs, the functions of *Pten* and *Lkb1* as tumor suppressors have been identified.^[^ ^12^, ^32^ ^]^ However, GEMMs are very costly and extremely time‐consuming, which limits their wide application. Traditional models such as tumor cell lines and PDXs, as well as recent cancer organoids, cannot recapitulate the entire process of multiple‐step tumorigenesis from normal cells into fully transformed malignancies. Here, based on recent breakthroughs in organoid culture, we report a new strategy for OPCMs of EC in mice. Referring to the numerous EC‐associated genetic abnormalities revealed by recent cancer genomics studies,^[^ ^6^, ^10^ ^]^ our approach can introduce any genetic abnormality you want into uterus organoids by CRISPR/Cas9 genome editing and gene overexpression techniques. Once orthotopically transplanted into recipient animals, these organoids are transformed into EC through a process similar to that observed in patients. This point differs from GEMMs, where all cells in the endometrial epithelium have the target gene mutation. This explains why *Pten* deletion could generate tumors in GEMMs,^[^ ^33^ ^]^ but using our strategy, TP organoids could not give rise to tumors in the observation period. Tumorigenesis using our strategy may require a longer period of time. With a similar strategy, OPCMs for lung cancer, gastric cancer, bladder cancer, and others have been generated.^[^ ^18b–e^ ^]^ These OPCMs could represent the pathologies of cancer at different stages, from dysplasia to full‐blown carcinoma with distal metastasis. Importantly, the resulting tumors can precisely recapitulate the pathology of their human counterpart. The limitation of the present EC strategy is that the recipient mice are immunodeficient mice, which cannot be applied to study tumor immune microenvironments and immunotherapy. Therefore, in order to expand the applicability of the model, we are optimizing the technical system to generate OPCMs of EC in immunocompetent mice. In fact, the small cell lung cancer models generated using a similar strategy are established in immunocompetent mice. On balance, compared to other models including GEMMs, this approach is very convenient and time‐saving. Our work emphasizes the application of a cancer‐modeling strategy with genetically engineered organoids in EC.

This new strategy might be especially important for the function of EC‐associated genes that have been difficult to be established using traditional methods. High‐throughput sequencing analysis for EC patient samples has demonstrated that phosphatidylinositol 3‐kinase (PI3K) pathway aberrations occur in more than 80% of endometrioid endometrial cancers.^[^ ^6^, ^8^ ^]^ Of particular interest, coordinate mutations of multiple PI3K pathway members are more common than predicted by chance, particularly since *PTEN* mutations frequently coexisted with *PIK3CA* or *PIK3R1*.^[^ ^10^ ^]^ However, despite the clinical data on coordinate mutations of these genes, there have been no genetics‐defined, primary, and orthotopic EC models available previously to study the mechanism. In this study, we applied a new strategy to create a series of EC OPCMs, including *Pten* mutation alone (TPM), *Pten* and *Pik3ca* co‐mutation (TPMCa) as well as *Pten* and *Pik3r1* co‐mutation (TPMR1) in the background of *Trp53* loss and *Myc* overexpression. We observed that in the premalignant stages, TPMR1 grew significantly faster, while TPMCa grew slower than TPM organoids. Furthermore, we found that in the malignant stages, the co‐mutation of *Pik3r1* or *Pik3ca* with *Pten* promoted EC tumorigenesis and shortened the survival time in mice. Importantly, TPMCa and TPMR1 tumors exhibited pathological features of endometrioid adenocarcinoma distinct from those of TPM tumors, mimicking the histological and molecular characteristics of human EC. Thus, we confirmed the function of *Pik3ca* and *Pik3r1* in EC. Our work provides experimental evidence for the frequent co‐mutation of *Pik3ca* or *Pik3r1* with *Pten*. Furthermore, this strategy provides a platform for testing the potential functions of the numerous other EC‐associated mutations in these models.

There are few treatment options available for patients with advanced or recurrent endometrial cancer, resulting in a poor prognosis.^[^ ^34^ ^]^ Recent advancements in genetic diversity and molecular classifications of this disease have created the potential to improve molecular‐targeted therapies and precision medicine. Fortunately, progress in EC patient‐derived organoid culture suggests a new opportunity to generate a precise drug screening and discovery tool. Although EC patient‐derived organoids fully recapitulate the molecular and pathological features, the establishment efficiency of ECOs depends heavily on the starting material. A previous study has shown that the success of the optimized conditions was only 40%, thereby limiting the application of patient‐derived organoids.^[^ ^35^ ^]^ In this study, we constructed a new drug screening pipeline with driver‐defined tumor organoids that exhibit genomic mutation‐specific drug responses. As an example, we performed a primary screening with 56 small molecule compounds on the four ECOs. To further test the in vitro inhibitory effects of drugs on these ECOs, we selected some candidate drugs and performed a second screening and validation experiment. In order to achieve rapid and high‐throughput drug screening, only one well was allocated for each drug at the concentration of 10 µM in the primary screening process, without any replicates being established. Therefore, the primary screening results may display a certain degree of irreproducibility. However, the results obtained from the validation experiments more accurately reflect the real‐world circumstances. Our screening and validation experiments revealed that TPK‐driven‐ESCC organoids displayed high resistance to most drugs, while dacomitinib significantly inhibited the growth of TPK ECOs. Notably, dacomitinib has been found to be effective against chemotherapy‐resistant HER2/neu gene‐amplified uterine serous carcinoma,^[^ ^36^ ^]^ but there are no more studies on dacomitinib and endometrial cancer. Further clinical trials testing these compounds in endometrial cancer patients would provide new insights. The strategy of the present study opens the possibility to test the novel drugs on driver‐defined ECOs in vitro, potentially yielding precise therapeutics for the treatment of this disease.

## 4 Experimental Section

### Mice

Mice were housed in a specific pathogen‐free animal facility at Sichuan University with autoclaved food, bedding, and water. All mouse experiments were performed in compliance with the Guide for the Care and Use of Laboratory Animals of Sichuan University and approved by the Animal Care and Use Committee of Sichuan University (approval number: 2021995A). CAG‐Cas9‐EGFP mice were purchased from Jackson Laboratories (Cat# JAX:026179, RRID: IMSR_JAX:026179) (female, 8–10 weeks old, ≈20 g weight). BALB/cA‐nu Mice (female, 8 weeks old, ≈20 g weight) used in the experiments were purchased from Beijing HuaFukang Biological Technology Co. Ltd.

### Cell Culture

HEK 293T cells (CRL‐1573) from ATCC were taken and cultured in DMEM containing 10% (vol/vol) fetal bovine serum and penicillin (100 U ml^−1^)/streptomycin (0.1 mg ml^−1^), placed at 37 °C, 5% CO_2_ cell incubator. The HEK 293T cell line was routinely tested for mycoplasma using PCR.

### Mouse Organoid Culture

The mouse uterus was removed and the mouse uterus body was separated and cut into 5 mm^3^ pieces. Samples were incubated with digestion buffer containing 1.0 mg ml^−1^ collagenase I (Gibco, 17100‐017) and 0.5 mg ml^−1^ collagenase IV (Gibco, 17104‐019) in DMEM/F12 (Gibco, C11330500BT) at 37 °C for 30 min. The mixture was filtered through a 100 µm cell strainer (JET BIOFIL, CSS‐013‐100) to obtain cell suspension, then centrifuged at 400× g for 5 min, and washed once with DMEM/F12. Cells were resuspended in ice‐cold Matrigel (BD, 354230) at a ratio of 1:20 (vol: vol), and 30 µl of the mixture was added to the bottom of 48 well plates to form a hemispherical shape. After solidification, 150 µl organoid expansion medium (ExM) was added to each well. The ExM was as follows: DMEM/F12 medium was supplemented with 2 mM GlutaMAX (Gibco, 35050079), 1× N2 supplement (Gibco, 17502001), 1× B27 supplement (Gibco, 17502001), 10 mM nicotinamide (Sigma, N0636), 1 mM N‐acetylcysteine (Sigma, A0737), 50 ng ml^−1^ human epidermal growth factor (PEPROTECH, 900‐M05), 100 ng mL^−1^ fibroblast growth factor‐10 (PerpoTech, 400–29A), 500 nM A83‐01 (Sellect, S7692), 10 µM Y27632 (Sellect, S6390), 100ng ml^−1^ noggin, and 10% conditioned medium containing Wnt3a (homemade) and R‐spondin (homemade). The medium was changed every 3 days. For passaging, organoids were dissociated using TrypLE Express (Gibco, 12605010) at 37 °C for 15 min. This process disrupted the spherical organoids into single cells which were then embedded in cold Matrigel.

### Human Organoid Culture

Endometrial cancer tissue samples were taken from patients undergoing endometrial cancer resection. The tumor was exfoliated from the specimen and cleaned three times with phosphate‐buffered saline (PBS) and then cut into 5mm^3^ pieces. The digestion and culture methods used were consistent with those described in the section on “mouse organoid culture”. The study was approved by the Ethical Research Committee of the West China Second Hospital (2017SZ0064). All experiments carried out with the full, informed consent of the subjects.

### Organoid Orthotopic Transplantation

Endometrial organoids were digested in TrypLE at 37 °C, then centrifuged to remove the supernatant and resuspended in Matrigel. After induction of anesthesia, the mice were incised at 0.8 cm on the lateral side of the abdomen, and the left uterus was clipped with tweezers. Then, 300000 cells were injected into the left uterus at a volume of 0.015 ml.

### Plasmid Constructs

Gene‐specific sgRNA oligos were cloned into the lentiviral vector V2TC, which bicistronically expresses sgRNAs and mCherry. sgRNAs (Table S1, Supporting information) were designed using CRISPR Design Tool (http://crispr.mit.edu/). The plasmids for expression of *Myc*, *Kras* G12D, and *Pik3ca* E545K were constructed into retroviral constructs including MSCV – *Myc*/*Kras* G12D/*Pik3ca* E545K – IRES – Luci2.

### Gene Editing and Efficiency Testing

After organoid dissociation into single cells with TrypLE, cells were resuspended with viral supernatant supplemented with 1: 1000 (v/v) polybrene in a 24‐well plate, centrifuged at 800 g for 1 h, and then incubated at 37 °C for 2 h. The solution was collected and centrifuged at 400 g for 5 min. The cells were resuspended in ice‐cold Matrigel and seeded in 48‐well plates for continued culturing. To validate the targeted mutations, genomic DNA was isolated from infected organoids, and the T7E1 (Vazyme, EN303‐01) assay was performed. Primer sequences were used to amplify mouse *Trp53*, *Pten* and *Pik3r1* are listed in Table S2, Supporting information. To compare the number and diameter of TPM, TPMR1, and TPMCa organoids after infection. In these three groups, the number of starting normal organoids was consistent, and the titers and volumes of virus used for infection were balanced.

### Bioluminescence Imaging

After being transplanted orthotopically with organoids, mice were periodically imaged to detect the luciferase fluorescence signal intensity with the IVIS Spectrum in vivo imaging system (PerkinElmer). The mice were anesthetized with isoflurane after 250 µl of 15mg ml^−1^ D‐luciferin, sodium salt (Bio Vision, #7903‐1G) in PBS intraperitoneally injected. 5 min later, the mice were placed into the instrument and imaging began.

### Western Blotting Analysis and Antibodies

Whole organoid lysates were extracted in RIPA buffer (Beyotime, Cat# P0013) supplemented with protease inhibitors (Beyotime, Cat# P1045). Then, lysate proteins were separated by 12% SDS‐PAGE gels and then transferred to PVDF membranes. Western blotting was performed using antibodies against PTEN (cell signaling, 9559 s) and MYC (Abcam, ab32072). Primary antibodies were applied at 1:1000 dilution in 5% non‐fatty milk in TBST and incubated overnight at 4 °C. HRP‐conjugated secondary antibodies were applied at 1:10 000 dilution. Images were developed by NCM ECL Ultra Reagent (NCM biotech).

### H&E and IHC Staining

Fresh tumor tissues were fixed in 4% PFA, dehydrated, embedded in paraffin, and cut into 5‐µm ‐thick sections. The sections were stained with hematoxylin for 1 min to identify the cell nucleus and eosin for 30 s to identify the cytoplasm. Finally, the sheets were dehydrated and sealed.

For immunohistochemistry, the sections were dewaxed, hydrated, and repaired in a microwave oven using 10 mM sodium citrate buffer. After the repair liquid was cooled, the film was placed in 3% Triton‐100 solution for 20 min for drilling. After drilling, the cell was washed with PBS three times, and endogenous peroxidase was removed. After blocking with 2% goat serum for 1 h at room temperature, the corresponding antibodies were added dropwise and incubated at 4 °C overnight. The following antibodies were used: estrogen receptor (Invitrogen, MA1‐80216), progesterone receptor (Abcam, ab101688), p63 (Abcam, ab735), cytokeratin 7 (Abcam, ab180598), KI67 (Abcam, ab16667), PTEN (Cell Signaling, 9559s), p53 (Proteintech, 10442‐1‐AP), MYC (Abcam, ab32072), PIK3CA (Abclonal, A0265), and PIK3R1 (HUABIO, ER64588). After washing three times with PBS, the reaction enhancement solution was added and incubated for 20 min, followed by washing with PBS three times, adding the secondary antibody, and incubating for 1 h at room temperature. DAB was used for signal detection (ZSGB‐BIO, ZLI‐9018), followed by staining with hematoxylin for 10 s and rinsing with water for 1 min. The sections were blocked with a Permount mounting medium. The stained images were scanned using a panoramic MIDI (3DHISTECH). Immunoreactivity was assessed using the following scoring approach: −, no immunoreactivity; +, moderate incomplete staining within > 10% of tumor cells or complete staining within ≤ 10% of tumor cells; ++, strong complete staining within > 50% of tumor cells.

### In Vitro Treatment

Endometrial cancer organoids were subjected to chemotherapy drugs and FDA Compound Library (Selleck). Drugs (Table S3, Supporting information) related to E&H, protein tyrosine kinase, and PI3K/Akt/mTOR pathways were selected. To assess drug response, 3000 single cells were cultured in a 96‐well plate embedded in 10 µl Matrigel. In the primary screening, each inhibitor was added to a well at a concentration of 10 µM after 24 h. Three days after treatment, the surviving organoids were quantified by Cell Counting Kit‐8 (MCE, HY‐K0301). The inhibition score for each drug was determined by comparing them to vehicle‐treated wells. In the second screening, the candidates were added into three replicate wells at a concentration of 10 µM. For validation, they were added at various concentrations (0, 0.156, 0.625, 2.5, 10, 40, and 160 µM) and the cell viability was measured after 72 h. The vehicle (DMSO; Sigma Aldrich, D8418) was used as a negative control.

### RNA‐Seq Analyses

Approximately 2 months after orthotopic (TPM, TPMCa, TPMR1, and TPK) transplantation, the mice were sacrificed to obtain their tumor tissue. Total RNA was extracted using TRIzol reagent (Applied Biosystems, 15596026) following the manufacturer's instructions. The bulk RNA‐seq data of the EC mouse models were sequenced using Illumina NovaSeq 6000 with 150‐bp paired‐end reads. Adapter, poly‐N, and low‐quality reads of raw sequencing data were removed by the company for downstream analysis.

Clean RNA‐seq data were aligned to the mm10 reference using STAR.^[^ ^37^ ^]^ Differential gene expression and transcripts normalization were analyzed using Deseq2.^[^ ^38^ ^]^ Genes with log2Foldchange > 1/←1 and *p*‐value < 0.01 were identified as significantly DEGs in the TPM versus normal analyses. Genes with log2Foldchange > 1/←1 and *p*‐value < 0.05 were identified as significant DEGs in the TPK or TPM versus TPMCA&TPMR1 analyses. Heatmap of DEGs and specific squamous marker genes were normalized by z‐scores and visualized by pheatmap (v1.0.12). DEGs in GO pathway enrichment analysis were performed by clusterProfiler (v3.14.3). GSEA identified significant similarities between two given groups by identifying prior–defined gene sets.^[^ ^39^ ^]^ GSEA was used for pathway enrichment analysis and was used to illustrate that the mouse endometrial organoids we constructed mimic human endometrium in molecular characteristics. The normalized expression of specific genes in the different EC models was visualized using ggpubr (v0.4.0) and ggplot2 (v3.3.5). The mutation sites of *Pik3ca* and *Kras* in the TPMCa and TPK tumors were exhibited using Integrative Genomics Viewer.^[^ ^40^ ^]^

Cell viability analysis of the first and second drug screening in each group was constructed by pheatmap (v1.0.12), and the common and unique response drug analyses in each group (cell viability ≤ 10%) were performed by upsetR.^[^ ^41^ ^]^

The RNA‐seq count data of normal mouse uterus tissue used in the molecular resemblance between the TPM EC mouse model and EC patients were obtained from GEO datasets GSE138103. The RNA‐seq count data of normal organoids of mouse endometrium and other tissues (lung, liver, esophagus, stomach, bladder) used in correlation analysis between normal mouse endometrium organoids and human endometrium were deposited in Table S4, Supporting Information.

RNA‐seq results files of patients with EC were downloaded from the TCGA‐UCEC cohort. GSEA was performed to clarify the similarities of the molecular features between the EC patients and EC mouse models. The OncoPrint and cooperation between the frequently altered genes such as *PTEN*, *TP53*, *MYC*, *PIK3R1*, and *PIK3CA* in EC patients were analyzed using cBioPortal and demonstrated using Vennerable (v3.1.0).^[^ ^42^ ^]^ The stats package was used for Fisher's test analyses of PIK3CA2 or PIK3R1 mutation events in EC patients. The Kaplan–Meier survival curves of TCGA‐UCEC patients were exhibited using survival (v3.2.7) and statistical powers were calculated by log‐rank test, the gene expression levels were determined using survminer (v0.4.8).^[^ ^43^ ^]^

The gene expression data of human normal endometrium and other normal tissue (lung, liver, esophagus, stomach, bladder) were derived from the paired normal tissue with the sample type of “solid tissue normal” in TCGA (https://www.cancer.gov/tcga). The gene signatures of “HUMAN UTERUS UP/DOWN” were generated by comparing the human normal endometrium to other normal tissues of TCGA, including lung‐LUAD, liver‐LIHC, esophagus‐ESCA, stomach‐STAD, and bladder‐BLCA. Compared with other TCGA normal tissues, the top 200 up‐regulated or down‐regulated significantly DEGs (*p*‐value < 0.05) in human normal endometrium were identified as “HUMAN UTERUS UP/DOWN” gene signatures.

All quantification and visualization of RNA‐seq data were performed on R v.3.6.

### In Vivo Treatment

In vivo experiments were performed on 8‐week‐old female nude mice. 7 × 10^4^ cells were injected in both flanks of nude mice. Tumors were measured with a caliper and treatments were started when the tumors reached a volume of 0.1 cm^3^, after randomization of mice into control and treatment groups. Nintedanib ethanesulfonate salt (50 mg kg^−1^; Selleck, Cat# S5234) was administered by oral gavage three times per week. Control mice were treated with H_2_O only. Tumor volumes were measured before each administration by caliper in subcutaneous models. Mice were sacrificed and analyzed at the indicated time points. After animal sacrifice, tumors were dissected and recorded.

### Statistical Analysis

The organoid diameter assay, tumor measurements, and in vitro treatment were analyzed for statistical significance using two‐sided unpaired parametric Student's *t*‐tests (Prism 8.0, GraphPad software). Statistical significance was denoted as * *p* < 0.05, **p < 0.01, *** *p* < 0.001, and **** *p* < 0.0001. The numbers of independent experiments, samples, or events are indicated in the figure legends. For the in vitro treatment experiments, all samples were randomly assigned to the vehicle or treatment groups. None of the data were excluded from this study.

## Conflict of Interest

The authors declare no conflict of interest.

## Author Contributions

J.C., S.D., and L.Z. contributed equally to this work. J.C., S.D., and L.Z. designed and performed the experiments, analyzed the data, and wrote the manuscript. J.C., S.D., F.N., C.S., Y.Q., Y.Li, M.W., M.Z., and H.P. carried out the experiments. L.Z., Y.P., X.C., X.P., and A.Z. performed bioinformatics analysis. Q.Z., S.Y., Z.L., Y.Liu, S.Z., and Z.L. provided resources and designed experiments. C.C. conceived the project and wrote the manuscript.

## Supporting information

Supporting Information

Supporting Information

Supporting Information

Supporting Information

Supporting Information

## Data Availability

All EC RNA‐seq data were deposited in the Gene Expression Omnibus database repository under accession number GSE203121. The EC genomic alteration data were downloaded from cBioPortal (https://genie.cbioportal.org/).
