# Supplementary material for: A New Type of Endometrial Cancer Models in Mice Revealing the Functional Roles of Genetic Drivers and Exploring their Susceptibilities
Source: Adv Sci (Weinh). 2023 Jun 20;10(24):2300383. doi: 10.1002/advs.202300383 (PMC10460855; doi:10.1002/advs.202300383)
Supplement: Supplementary file 1 — Supporting Information [file ADVS-10-2300383-s001.pdf]

ADVANCED SCIENCE Open Access

Supporting Information

for *Adv. Sci.*, DOI 10.1002/advs.202300383

A New Type of Endometrial Cancer Models in Mice Revealing the Functional Roles of Genetic Drivers and Exploring their Susceptibilities

Jingyao Chen, Siqi Dai, Lei Zhao, Yiman Peng, Chongen Sun, Hongling Peng, Qian Zhong, Yuan Quan, Yue Li, Xuelan Chen, Xiangyu Pan, Ailing Zhong, Manli Wang, Mengsha Zhang, Shengyong Yang, You Lu, Zhong Lian, Yu Liu, Shengtao Zhou, Zhengyu Li, Feifei Na and Chong Chen**

Supplementary Figures

Supplementary Figure 1, related to Figure 1

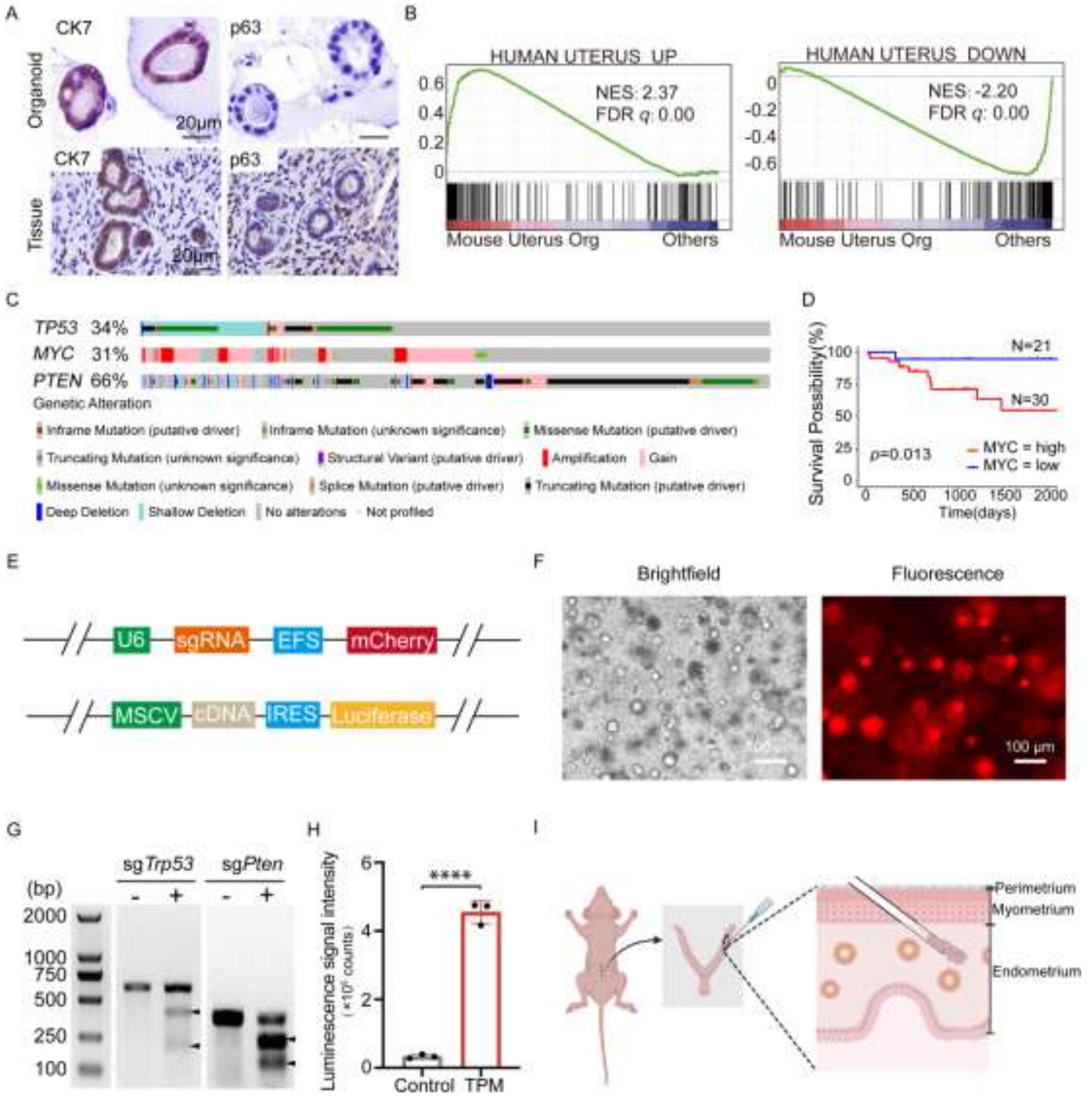

Supplementary Figure 1. Primary and orthotopic endometrial cancers initiated with genome-editing endometrial organoids in mice.

A. IHC stainings of normal uterus organoids and tissue. Scale bar, 20 μm.

B. GSEA showing positive enrichment of the HUMAN UTERUS UP (NES= 2.37, FDR *q*= 0.00) and negative enrichment of the HUMAN UTERUS DOWN (NES= -2.20, FDR *q*= 0.00) in mouse uterus organoids, compared to the organoids of other organs (lung, liver, esophagus, stomach, bladder). NES, normalized enrichment score; FDR, false discovery rate. UP/DOWN gene signatures, top 200 DEGs (*p*-value < 0.05) of up-regulated or downregulated in human normal uterus.

C. OncoPrint showing the variation frequencies of *TP53*, *MYC* and *PTEN* in 1954 EC samples from the cBioPortal dataset.

D. The Kaplan-Meier survival curves of *TP53* and *PTEN* variation patients with high or low expression levels of *MYC* in the TCGA-UCEC cohort. Statistical significance was determined by Log-rank test.

E. Schematic of sgRNA (top) and *Myc* cDNA (bottom) expression plasmid used in these experiments.

F. Bright-field and fluorescence images of mouse uterus organoids transduced by viral vectors. Scale bar, 100 µm.

G. T7 endonuclease 1 (T7E1) assays on *Trp53* and *Pten* using infected organoids from F. Cleaved bands were pointed by arrowheads.

H. Luminescence signal intensity of control and TPM organoids. Data are shown as mean ± SD. ****, $p < 0.0001$, calculated by two-sided Student’s t-test.

I. Schematic diagram showing the method of detailed orthotopic transplantation.

Supplementary Figure 2, related to Figure 2

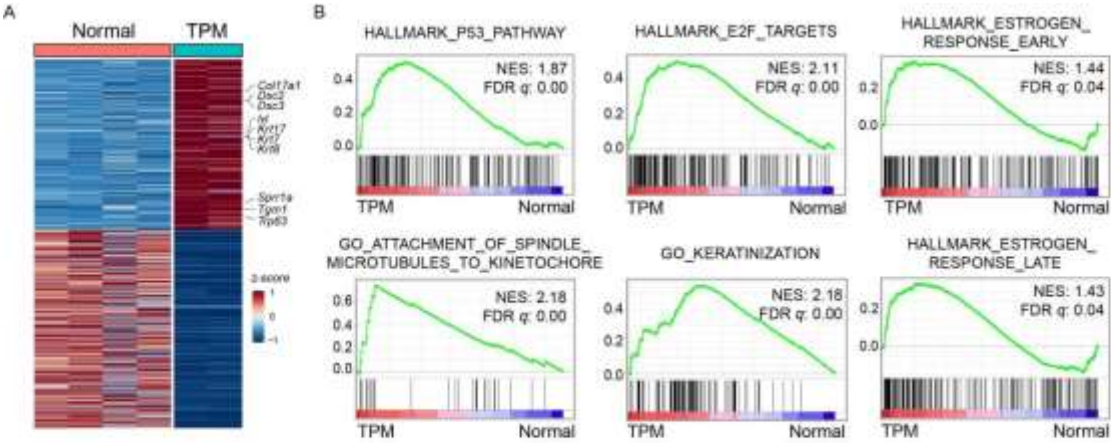

Supplementary Figure 2. Disruption of *Trp53* and *Pten*, together with *Myc* overexpression generated endometrial adenocarcinoma with squamous differentiation.

A. Heatmap showing the significantly upregulated and downregulated genes in the TPM mice tumor tissues compared with normal mice tissues. Squamous and adenoid marker genes were indicated by arrows (Normal: n = 4 mice; TPM: n = 2 mice).

B. GSEA showing positive enrichments of the HALLMARK_P53_PATHWAY (NES= 1.87, FDR *q*= 0.00), the HALLMARK_E2F_TARGETS (NES= 2.11, FDR *q*= 0.00), the HALLMARK_ESTROGEN_RESPONSE_EARLY (NES= 1.44, FDR *q*= 0.04), the GO_ATTACHMENT_OF_SPINDLE_MICROTUBULES_TO_KINETOCHORE (NES= 2.18, FDR *q*= 0.00), the GO_KERATINIZATION (NES= 2.18, FDR *q*= 0.00) and the HALLMARK_ESTROGEN_RESPONSE_LATE (NES= 1.43, FDR *q*= 0.04) signatures in the TPM mice tumor tissues compared with normal mice tissues.

Supplementary Figure 3, related to Figure 3

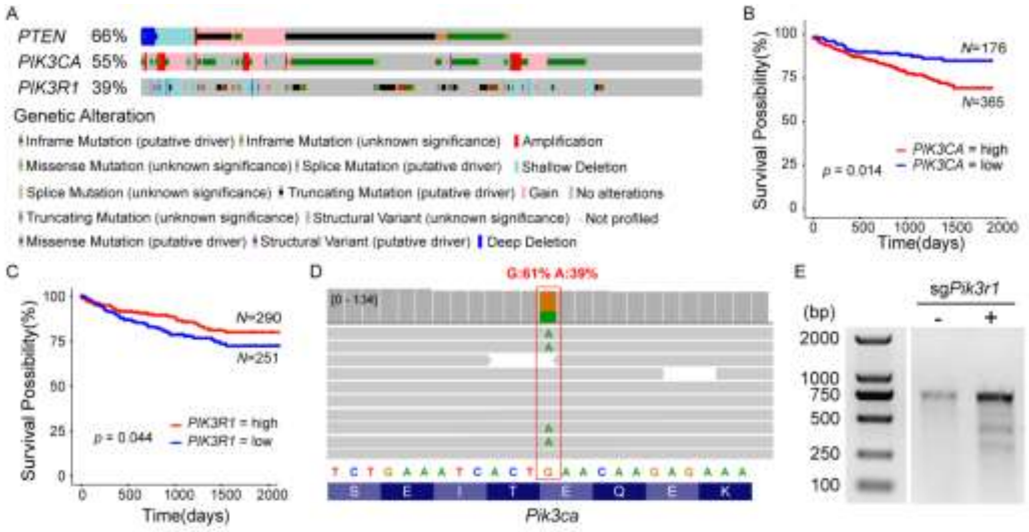

Supplementary Figure 3. *Pik3ca* and *Pik3r1* mutations accelerated the progression of TPM tumors.

A. OncoPrint showing the variation frequencies of *PTEN*, *PIK3CA* and *PIK3R1* in 1954 EC samples from the cBioPortal dataset.

B. The Kaplan-Meier survival curves of patients with high or low expression levels of *PIK3CA* in the TCGA-UCEC cohort. Statistical significance was determined by Log-rank test.

C. The Kaplan-Meier survival curves of patients with high or low expression levels of *PIK3R1* in the TCGA-UCEC cohort. Statistical significance was determined by Log-rank test.

D. Integrative Genomics Viewer exhibiting the mutation site of *Pik3ca* in the TPMCa tumor.

E. T7 endonuclease 1 (T7E1) assays on *Pik3r1* using the infected organoids.

Supplementary Figure 4, related to Figure 4

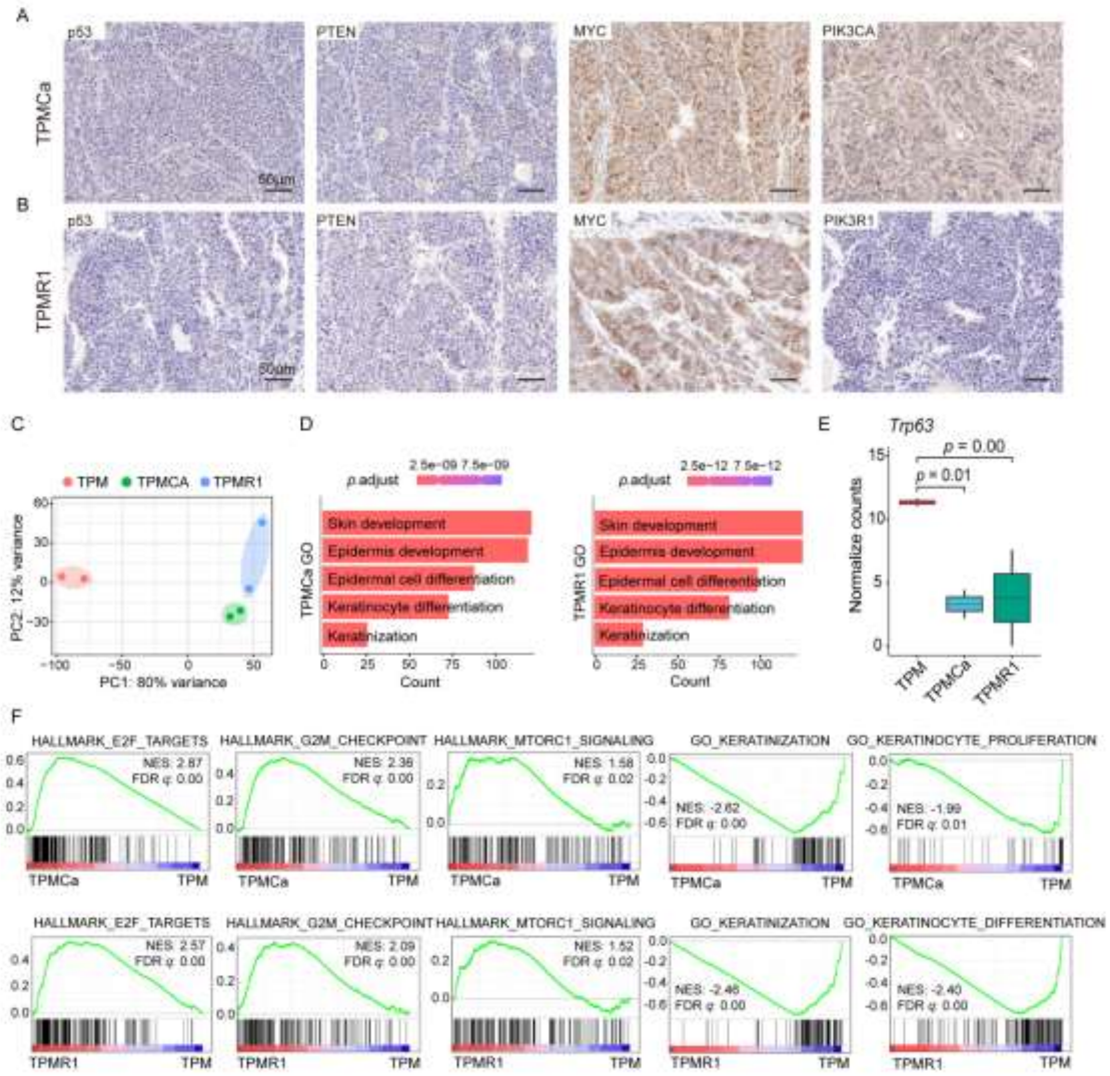

Supplementary Figure 4. *Pik3ca* and *Pik3r1* mutations were dominant in the endometrial adenocarcinoma.

A. Representative pictures showing IHC stainings of p53, PTEN, MYC and PIK3CA in TPMCa tumor. Scale bar, 50 µm.

B. Representative pictures showing IHC stainings of p53, PTEN, MYC and PIK3R1 in TPMR1 tumor. Scale bar, 50 µm.

C. The dot plot showing principal component analysis result of RNA-seq data from the TPM, TPMCa and TPMR1 tumors.

D. Gene Ontology enrichment plot of the downregulated genes in the TPMCa and TPMR1 mice tumor tissues compared to the TPM mice tumor tissues.

E. Box plot showing the expression level of *Trp63* in tumor tissuess of the TPM, TPMCa, and TPMR1 mice.

F. GSEA showing the enrichments of the HALLMARK_E2F_TARGETS (TPMCa: NES= 2.87, FDR q= 0.00; TPMR1: NES= 2.57, FDR q= 0.00), the HALLMARK_G2M_CHECKPOINT (TPMCa: NES= 2.36, FDR q= 0.00; TPMR1: NES= 2.09, FDR q= 0.00), the HALLMARK_MTORC1_SIGNALING (TPMCa: NES= 1.58, FDR q= 0.02; TPMR1: NES=

1.52, FDR *q*= 0.02), the GO_KERATINIZATION (TPMCa: NES= -2.62, FDR *q*= 0.00; TPMR1: NES= -2.46, FDR *q*= 0.00), the GO_KERATINOCYTE_PROLIFERATION (TPMCa: NES= -1.99, FDR *q*= 0.01) and the GO_KERATINOCYTE_DIFFERENTIATION (TPMR1: NES= -2.40, FDR *q*= 0.00) pathways in the TPMCa or TPMR1 mice tumor tissues compared with the TPM mice tumor tissues.

Supplementary Figure 5, related to Figure 5

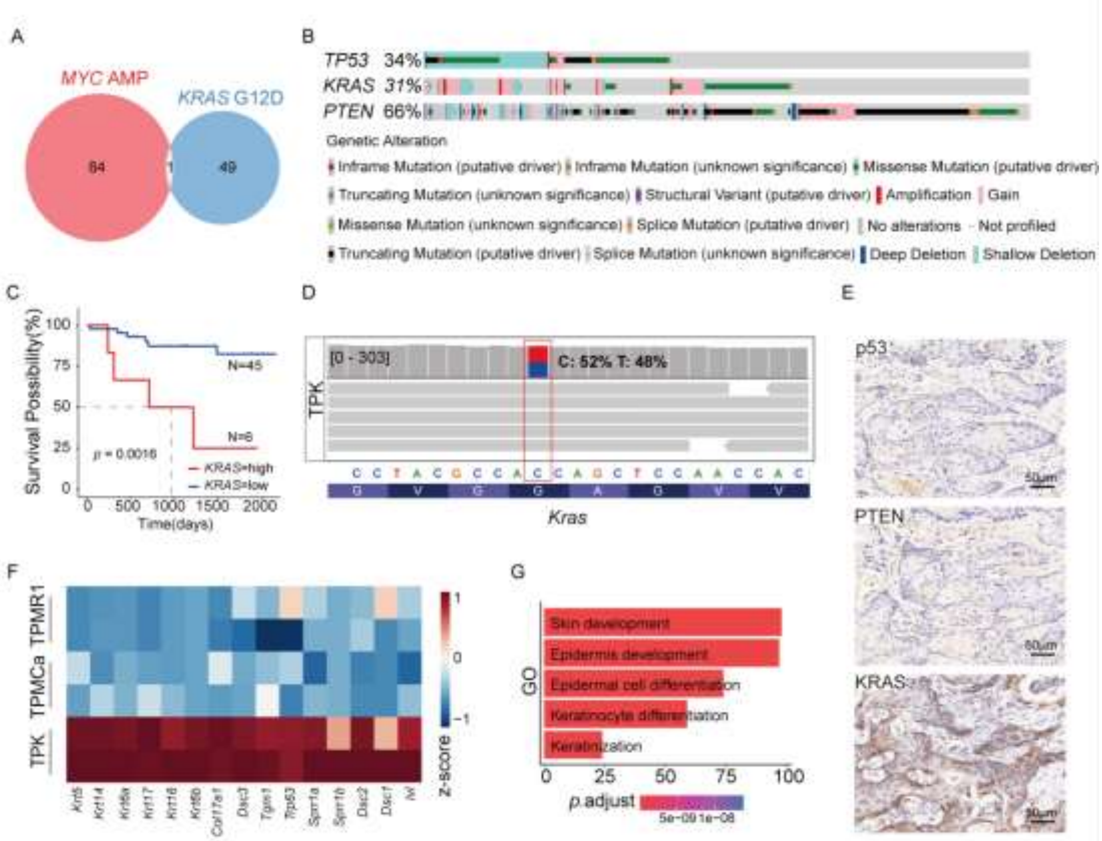

Supplementary Figure 5. ***Kras*** **G12D mutation led to endometrial squamous cell carcinoma in mice.**

A. Venn diagram showing the intersection of the human EC patients with *MYC* amplification and *KRAS* G12D mutation. EC patients data were analyzed from the cBioPortal dataset.

B. OncoPrint showing the variation frequencies of *TP53*, *KRAS* and *PTEN* in 1954 EC samples from the cBioPortal dataset.

C. The Kaplan-Meier survival curves of *TP53* and *PTEN* variation patients with high or low expression levels of *KRAS* in the TCGA-UCEC cohort. Statistical significance was determined by Log-rank test.

D. Integrative Genomics Viewer exhibiting the mutation site of *Kras* in the TPK tumor.

E. Representative pictures showing IHC stainings of p53, PTEN and KRAS in TPK tumor. Scale bar, 50 µm.

F. Heatmap showing the expression levels of squamous marker genes between the TPK, TPMCa and TPMR1 mice tumor tissues. (TPK: n = 2 mice; TPMCa: n = 2 mice; TPMR1: n = 2 mice).

G. Gene Ontology enrichment plot of the upregulated genes in the TPK mice tumor tissues compared to the TPMCa and TPMR1 mice tumor tissues.

Supplementary Figure 6, related to Figure 6

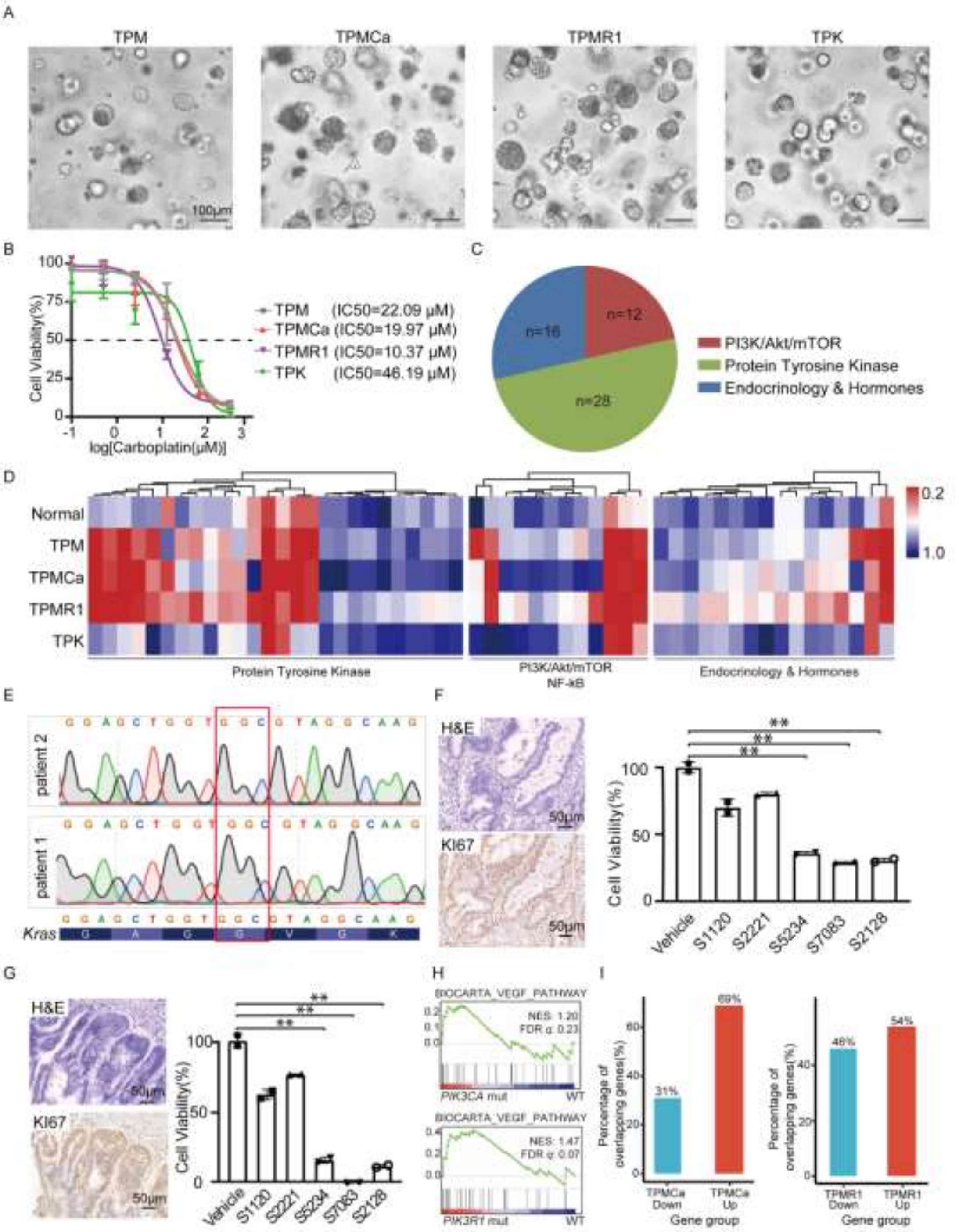

Supplementary Figure 6. Precision EC models for drug screening

A. Bright-field images of the TPM, TPMCa, TPMR1, and TPK EC organoids. Scale bar, 100μm.

B. Drug dose-response curve showing cell viability of the TPM, TPMCa, TPMR1, and TPK EC organoids in response to the treatment of carboplatin. IC50 was shown. n = 3.

C. Statistics of targeted pathways for drug screening.

D. Heatmap showing the relative survival of EC organoids treated with 56 drugs from the FDA drug library at concentrations of 10 μM. The numbers in the color block indicated the cell viability.

E. Gene sequencing peak map of *KRAS* in patient 1 and patient 2 with EC. The box indicates the the 12th amino acid and its codon in *KRAS* gene.

F. H&E (top) and IHC (bottom) staining of KI67 in patient 1 tumor. Scale bar, 50 μm (left). Cell viability of patient 1-derived organoids treated with S1120, S2221, S5234, S7083 and S2128, comparing to those of vehicle. Data shows the means ± SD. $^{**}p < 0.01$ (right).

G. H&E (top) and IHC (bottom) staining of KI67 in patient 2 tumor. Scale bar, 50 μm (left). Cell viability of patient 2-derived organoids treated with S1120, S2221, S5234, S7083 and S2128, comparing to those of vehicle. Data shows the means ± SD. $^{**}p < 0.01$ (right).

H. GSEA showing the enrichments of the BIOCARTA_VEGF_PATHWAY in human EC with *PIK3CA*/*PIK3R1* mutation (NES=1.20, FDR q=0.23; NES=1.47, FDR q=0.07) compared with WT.

I. Bar plot showing the proportion of BIOCARTA_VEGF_PATHWAY genes in the TPMCa/R1 mice compared with TPM (|log2FoldChange| > 0).
